# Management of asthma in primary care in the changing context of the COVID-19 pandemic: a qualitative longitudinal study with patients

**DOI:** 10.3399/BJGP.2022.0581

**Published:** 2023-07-11

**Authors:** Marta Santillo, Sarah Tonkin-Crine, Kay Wang, Christopher C Butler, Marta Wanat

**Affiliations:** Nuffield Department of Primary Care Health Sciences, University of Oxford, Oxford.; Nuffield Department of Primary Care Health Sciences, University of Oxford, Oxford.; Nuffield Department of Primary Care Health Sciences, University of Oxford, Oxford.; Nuffield Department of Primary Care Health Sciences, University of Oxford, Oxford.; Nuffield Department of Primary Care Health Sciences, University of Oxford, Oxford.

**Keywords:** asthma, COVID-19 pandemic, longitudinal interviews, primary care, qualitative research

## Abstract

**Background:**

The COVID-19 pandemic dramatically affected asthma monitoring in primary care, but exploration of patients’ views and their experiences of managing their asthma and seeking help from primary care during the pandemic has been limited.

**Aim:**

To investigate patients’ experiences of asthma management in the community during the COVID-19 pandemic.

**Design and setting:**

A qualitative longitudinal study using semi-structured interviews with patients from four GP practices across diverse regions including Thames Valley, Greater Manchester, Yorkshire, and North West Coast.

**Method:**

Interviews were undertaken with patients with asthma, who were usually managed in primary care. The interviews were audiorecorded, transcribed, and analysed using inductive temporal thematic analysis and a trajectory approach.

**Results:**

Forty-six interviews were conducted with 18 patients over an 8-month period that covered contrasting stages of the COVID-19 pandemic. Patients felt less vulnerable as the pandemic subsided, but the process of making sense of risk was dynamic and influenced by multiple factors. Patients relied on self-management strategies, but felt that routine asthma reviews should still have been conducted during the pandemic and highlighted that they had limited opportunities to discuss their asthma with health professionals. Patients with well-controlled symptoms felt that remote reviews were largely satisfactory, but still thought face-to-face reviews were necessary for certain aspects, such as physical examination and patient-led discussions of sensitive or broader issues associated with asthma, including mental health.

**Conclusion:**

The dynamic nature of patients’ perception of risk throughout the pandemic highlighted the need for greater clarity regarding personal risk. Having an opportunity to discuss their asthma is important to patients, even when access to face-to-face consultations in primary care is more restricted than usual.

## INTRODUCTION

The COVID-19 pandemic dramatically affected asthma monitoring in primary care.^1^ Some routine asthma reviews were postponed or conducted remotely,^2^ which meant that some important elements for optimal management of asthma, such as checking inhaler technique^3^ or reviewing medication,^4^ could either not take place or became more difficult to implement.^2^ The limited access could have been particularly impactful for patients with severe asthma, some of whom go unrecognised in primary care,^5^ and there may have been limited opportunities to review the validity of recorded diagnoses.^6^ Although a backlog of reviews has been decreasing, additional priorities — including the COVID-19 vaccine rollout — continue to impinge on the completion of asthma reviews.^7^

Self-management of asthma — defined as the tasks that individuals with asthma may do to live with the condition, including having the confidence to deal with both the medical and emotional management of their condition^8^ — improves asthma control.^9^ In the context of the pandemic, patients may have been uncertain about how or when to contact their GP, which, in turn, may have led to poorer outcomes for them.^1^ There were also uncertainties about whether patients with asthma were at increased risk from COVID-19.^10^^–^^12^ This was evidenced by patients with severe asthma being advised to shield in the initial stages of the pandemic,^11^ and was further reflected in uncertainties related to whether patients with asthma should be offered a COVID-19 vaccine, as well as additional boosters, as a priority, which may have created confusion for patients. Further, anxiety and depression are more commonly reported in patients with asthma and are associated with worse clinical outcomes;^13^^,^^14^ the pandemic may have exacerbated these conditions,^15^ which, in turn, may have affected patients’ adherence to medication and asthma control,^16^ as well as limiting the opportunities to review the validity of recorded diagnoses.

Previous qualitative studies focused on primary healthcare professionals’ experiences of delivering remote care during the pandemic, highlighting a number of challenges;^2^^,^^17^^,^^18^ in contrast, the evidence on patient experiences of managing their asthma has been limited. The studies undertaken so far have, mainly, focused on patients’ views and reported patterns of requesting medication during the pandemic,^19^ patients’ and healthcare professionals’ beliefs about asthma and COVID-19,^16^ caregiver experiences of managing childhood asthma,^20^^–^^22^ and the effects of having asthma on mental health.^15^^,^^23^^,^^24^ However, to the authors’ knowledge, patients’ views and experiences of managing their condition and seeking help from primary care during the pandemic have not been explored. As diagnosis and asthma monitoring are mainly managed in primary care, which continues to undergo substantial changes as a result of the COVID-19 pandemic,^17^ understanding how patients’ experiences may change over time is crucial. This study aimed to fill this important gap by longitudinally exploring patients’ views and experiences of asthma monitoring in primary care during the COVID-19 pandemic, with the aim of identifying barriers and facilitators to asthma management in the context of the pandemic and beyond.

**Table:** How this fits in

The COVID-19 pandemic had a significant impact on asthma monitoring in primary care, but there has been limited evidence on patients’ views and their experiences of managing their asthma. Through longitudinal interviews with patients over 8 months, the authors explored how patients tried to make sense of the effect asthma would have on their risk of complications from COVID-19, how they engaged with self-management strategies, and what their needs and experiences were in relation to routine asthma reviews. The study highlights what patients with asthma may find helpful when managing their condition in times of more-limited contact with health professionals, and the aspects with which they may struggle; the role of primary care in addressing these is highlighted, and may also be relevant to the management of other long-term conditions.

## METHOD

### Design

This was a qualitative longitudinal study using serial interviews with each patient; the qualitative study design is best suitable for understanding continuity and changes in patient views, and how shifting context can influence care provision and subsequent patient experience.^25^^–^^27^

### Patient and Public Involvement

Throughout the study, the authors worked with a patient and public involvement (PPI) panel of four asthma patients. These patients were recruited via Asthma UK by responding to an advertisement placed in the Asthma UK newsletter. The panel was involved throughout the whole study cycle and advised on design of the study, patient-facing documents, analysis, and summary of findings sent to the participants.

### Sampling and recruitment

With help from three Clinical Research Networks, participants were recruited from four GP practices serving diverse regions across the UK including: Thames Valley, Greater Manchester, Yorkshire, and North West Coast; these were selected based on their geographical area, size, and deprivation and diversity indices. Each practice identified and invited approximately 50 patients aged >18 years, with ‘active asthma’ (defined as a coded diagnosis of asthma and having had a prescription for at least one asthma medication in the previous year), who had had a review in the previous 3 months or were due a review in the following 3 months. The search was carried out by the practice staff. Each practice contacted the participants in the way they usually contact them (for example, by email or text message). Participants interested in learning more about the study were asked to contact the research team. Interested participants were provided with a participant information leaflet and a written copy of the verbal consent form.

The authors aimed to recruit a maximum variation sample, based on age, sex, time since diagnosis, self-reported number of asthma exacerbations in previous 12 months, and use of asthma action plans. Interested participants contacted the study team directly and were given the study information.

### Data collection

Longitudinal interviews were conducted at 3-month intervals over 8 months (December 2021 to July 2022). Box 1 shows brief contextual information related to the timing of the interviews, which were conducted across different time points to capture changes to asthma management influenced by external factors related to the COVID-19 pandemic (such as practices being asked to prioritise areas of care based on their own judgement).^28^

**Box 1.** Contextual information related to timing of interviews

**Interview wave**	**Date range**	**Extent of restrictions and other key policies**	**Key events related to asthma and primary care**
**Time 1**	8 December 2021 to 31 January 2022	New variant of COVID-19 (Omicron) is confirmed; tighter travel restrictions are brought in; ‘plan B’ restrictions are implemented (working from home, compulsory face masks); vaccine booster programme becomes a priority; 19 January was the end of plan B	COVID-19 vaccination programme is accelerated; QOF requirements change; practices are asked to prioritise areas of care, based on their judgement^28^
**Time 2**	7 March 2022 to 10 April 2022	Restrictions are lifted; free COVID-19 testing ends on 1 April 2022; people with COVID-19 symptoms are advised to stay at home (but this is no longer compulsory)	QOF recommences April 2022^23^
**Time 3**	13 June 2022 to 7 July 2022	No restrictions	No key events

*QOF = Quality and Outcomes Framework.*

Two expert qualitative researchers shared the data collection, and each researcher carried out all interviews with the same participant throughout. Semi-structured telephone interviews were conducted using a topic guide covering subjects of interest, while allowing individuals to express their own thoughts and to discuss topics of most importance to them. All interviews were audiorecorded and transcribed verbatim.

Informed consent was obtained verbally prior to interview and a written record was retained. Participants were assured that they could withdraw from the study at any time, and were offered £20 in high street vouchers for each interview.

### Analysis

**Figure 1.**
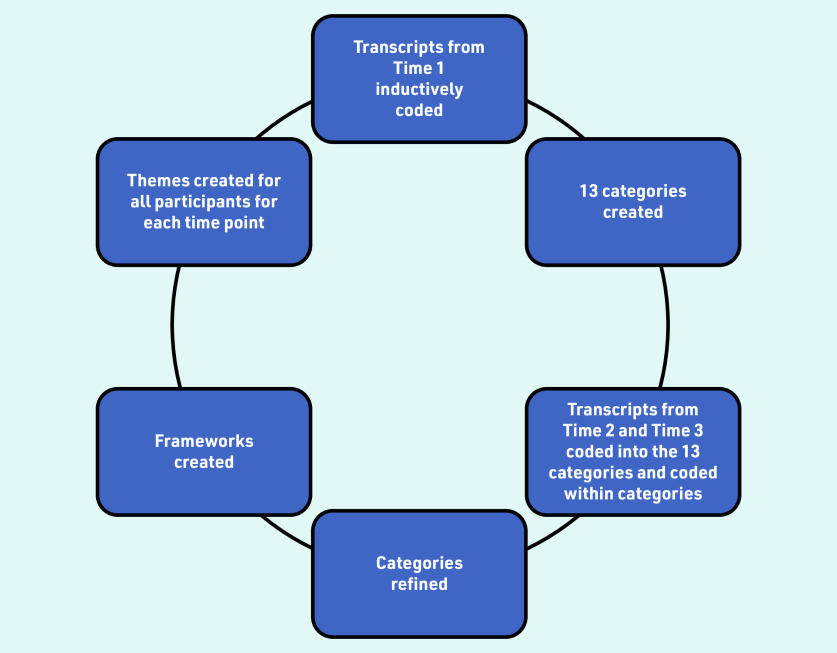
*Overview of the steps involved in the analysis.*

Data collection and analysis took place concurrently. Data were analysed using an inductive temporal thematic analysis^25^ and trajectory approach.^29^ Figure 1 provides an overview of the process. The two researchers who conducted the interviews read all transcripts from Time 1, and inductively coded them and grouped them into 13 categories. The analysis of interviews at Time 2 and Time 3 was guided by these categories; this meant that transcripts were deductively coded into these categories but, within each category, data were inductively coded to maintain familiarisation.

The second phase of analysis involved focusing on temporal aspects, with the aim of identifying key similarities and differences across time points and participants. To aid this, two frameworks summarising data across three time points were created: per participant and per category. These frameworks are helpful alongside thematic analysis to enable visualisation of findings over time.^25^ The participant framework was used to identify changes and continuity in views and experiences over time for each individual patient, while the category-based framework was used to identify similarities and differences in relation to each specific category across time points for all patients;^25^ this allowed the categories to be grouped into themes. The analysis was an iterative process, going back and forth between time points, categories, and themes.

Four patients with asthma, who were recruited via Asthma UK, were involved throughout the study. The ongoing analysis from each time point was discussed with them as the study progressed. A summary of the results was also shared with all interview participants. The authors adhered to the Consolidated Criteria for Reporting Qualitative Research reporting guideline.^30^

## RESULTS

In total, 46 semi-structured interviews were conducted with 18 patients over an 8-month period. Interviews lasted a median of 25 (range 20–40) minutes each. Table 1 provides a summary of the patients’ key characteristics and Box 2 summarises the key findings at each time point. Three themes were identified:

- making sense of one’s vulnerability to COVID-19;
- views on asthma reviews during the pandemic; and
- the role of self-management strategies during the pandemic.

**Table 1.** Summary of patients’ (*n* = 18) key characteristics

**Characteristic**	***n* (%)^a^**
**Sex**	
Female	9 (50)

**Mean age, years (SD)**	50 (18)

**≥1 self-reported number of asthma exacerbations in the previous 12 months**	6 (33)

**Patients completing interviews**	
Time 1	18 (100)
Time 2	14 (78)
Time 3	14 (78)

**Timing of last asthma review**	
Before the pandemic	6 (33)
During the pandemic	12 (67)

**Infected with COVID-19 before and/or during the study period**	8 (44)

**Received information from NHS advising on their risk**	4 (22)

a *Unless otherwise stated. SD = standard deviation.*

**Box 2.** Summary of findings by theme

**Theme**	**Summary**	**Key findings**		
		**Time 1**	**Time 2**	**Time 3**
**Making sense of one’s vulnerability to COVID-19**	Participants tried to make sense of their risk related to having asthma and experiencing consequences of COVID-19	Participants felt at higher risk of developing severe illness or complications compared with people without asthma Perceptions of risk affected by external factors (for example, letters from the NHS) or internal factors (based on perceived asthma severity or symptom control) Levels of activity (for example, going out) affected by perception of risk	Participants continued to feel at higher risk compared with people without asthma, but perceptions of risk dynamically changed depending on individual circumstances Participants continued life with some levels of restriction but to a lesser extent than at Time 1	Participants felt less at risk than before, including those who felt significantly vulnerable Participants resumed previous activities, including those they had previously avoided (for example, taking a holiday)
**Views on asthma reviews during the pandemic**	Participants shared their views and experiences of having, and not having, access to asthma reviews	Participants differed in their acceptance of the lack of reviews based on perceived asthma control, symptom severity, and a relationship with their GP practice Participants saw telephone reviews as convenient but more rigid than face-to-face reviews, which were deemed a ‘gold standard’	Participants highlighted the need for a review linked to their worries of not having had a check-up for a long time	Participants, including those with well-controlled asthma, expressed concerns about not having had a review Those who had been offered asthma reviews highlighted the need for, and expectations of, a more-comprehensive review after not having had one for a long time
**The role of self-management strategies during the pandemic**	Participants discussed which self-management strategies they used during the pandemic and what helped them to manage their asthma	Participants relied on known self-management strategies and highlighted their importance during the pandemic Participants highlighted worries about problems accessing inhalers and managing their mental health	Participants continued with their self-management strategies Participants with experience of infections sought help from their general practice	Participants continued with their self-management strategies, highlighting the need to prioritise their mental health

### Making sense of one’s vulnerability to COVID-19

#### Time 1

Overall, patients perceived that they were at higher risk of developing severe illness or complications from COVID-19, or experiencing more-severe illness from COVID-19 compared with people without asthma. For some patients, their perception of their risk was triggered by letters and communication from the NHS, informing them that they were clinically vulnerable or in a higher-risk category. However, receiving information or being reassured by their GP practice that they did not need to have the COVID-19 vaccine earlier than other people in their age group was interpreted by patients as confirmation of a low-risk status. Patients highlighted that they were not sure what the letters meant, with a minority seeking further information from government, and other, websites:

*‘I wondered how they knew how fatal it would be if I got it because the letter was written in a way that if I get it, I would die and* […] *I had questions of* […] *how do I keep myself as safe* […] *I relied heavily on Asthma UK’s website and they had a lot of advice on there for people that were shielding.’*

(Participant [P]16, aged 20 years, asthma not well controlled)

In contrast, the majority of patients, who had not received letters, tried to make sense of the risk themselves. The extent of risk seemed to be linked to their perceived asthma severity or symptom control, with patients who considered their asthma to be severe being more concerned about the risk that COVID-19 might make them more severely unwell than people who were otherwise healthy:

*‘If I look at the stats, COVID is pretty mild for most people but, on top of something else like severe asthma, that can be the thing that gets you.’*

(P5, aged 61 years, asthma well controlled)

Consequently, the more patients felt at risk, the more they seemed to restrict their activities and incorporate infection-control behaviours, such as wearing a mask or not going out:

*‘I think I was more scared shall we say given that everything I was hearing about COVID, that it attacks a respiratory system, so I felt particularly vulnerable and likely to be quite careful that’s why I’ve been taking all the measures.’*

(P2, aged 69 years, asthma well controlled)

#### Time 2

Overall, patients seemed to continue to see their risk as higher than that of people without asthma, but patients’ individual experiences seemed to be affected by a variety of factors that were, often, dynamically changing. Factors contributing to a lower sense of risk included:

- stepping up medication;
- perceived good asthma control; or
- contracting COVID-19 and experiencing little impact on their asthma.

In contrast, the following seemed to increase patients’ sense of risk:

- uncontrolled symptoms;
- knowing people without asthma who experienced severe consequences of COVID-19;
- worries about new variants emerging;
- looking after an older relative; or
- living in an area where cases were still high.

A number of these patients felt that restrictions were lifted prematurely:

*‘I know people are getting it and it’s very mild, but there’s always a new strain on the horizon, isn’t there, so I still feel very vulnerable. I think a lot of people think, “Well, that’s it. It’s over”, and it isn’t necessarily that’s it over.’*

(P6, aged 69 years, asthma not well controlled)

Consequently, patients continued with some infection prevention and control measures that went beyond what the rules were at the time but were, in general, less strict than those in place at Time 1.

However, patients also often highlighted the difficulty of knowing how at risk they were:

*‘They obviously thought I was vulnerable in the first place because I had asthma;* […] *Well, I’m not too sure what scale of vulnerable* [laughs] *I’m on between nought and a hundred. If it’s 48%, therefore it’s not quite as vulnerable as it could be, that’s still pretty vulnerable!’*

(P3, aged 73 years, asthma not well controlled)

The lack of information about the level of risk was still of importance, which made it difficult for patients to know whether they should engage in certain activities (for example, seeing a large group of people):

*‘I feel slightly nervous about them* [lifting restrictions] *because obviously the normal day-to-day person doesn’t particularly have to be as worried at making sure that if they got COVID that they have to make sure that they have people on standby and medication on standby just in case. So I think it makes me more cautious of people who I know aren’t vulnerable because they are more flippant in their actions, I think.’*

(P16, aged 20 years, asthma not well controlled)

#### Time 3

By the time of the third interview, the majority of patients felt, overall, less at risk than before; even participants who had previously felt at substantial risk saw themselves as less vulnerable. This seemed to be closely linked to:

- wanting to return to feeling normal;
- seeing the current variant as causing milder symptoms;
- feeling reassured by having an additional booster; or
- experiencing mild symptoms when contracting COVID-19.

In contrast, worrying about high numbers of cases locally or getting an invitation letter for an extra booster because of being vulnerable seemed to act as a reminder that they may still be at risk:

*‘As time’s gone on, I’ve not felt as at risk but when I got the message to go for the fourth one* [vaccine], *I thought well I’m still on that vulnerable list and you do realise then you are vulnerable.’*

(P6, aged 69 years, asthma not well controlled)

Participants also described reducing the use of face masks or taking a ‘big risk’, such as going on holiday for the first time. The majority of patients also reported waiting for the official guidance on whether to have the second booster and some wondered whether the lack of information about the next booster meant they were not at risk:

*‘The only reason I think is, and I’m one hundred per cent for vaccination, it’s just because there was such a big research over the third and how the third was so important and how the third was, this is your extra protection that not everybody else needs, and now I’m a bit more like, oh, there’s a fourth now, like there’s either something that’s not being publicly displayed potentially about what they know about COVID and vulnerable people or maybe it is just being given because they can see vulnerable people potentially need more of a comfort, that maybe that’s also why they’re doing it. I’m not sure and I’ve not read into it yet because I’ve not been offered it but I don’t know, I’m just a bit more cautious about the fourth one I think.’*

(P16, aged 20 years, asthma not well controlled)

### Views on asthma reviews during the pandemic

#### Time 1

Overall, patients expressed uncertainty of what kind of support they could expect from general practice, given the pandemic. Among patients who had not had an asthma review, there seemed to be variation in the extent to which they accepted it; patients with more-severe or poorly controlled asthma, and patients with more recently diagnosed asthma highlighted the lack of reviews more, as they were keen to receive advice, have their medication checked, or get reassurance:

*‘I would like to have it* [review] *soon just because I’ve been on this medication for quite a while and I’d just like to see if my asthma’s improved since my last review; if I’ve got worse then I need to be upping it.’*

(P16, aged 20 years, asthma not well controlled)

In contrast, patients who reported having a good relationship with their GP practice seemed to accept the lack of review as they felt confident that they would know who to contact if their asthma got worse. Of note is that a minority of patients received more-frequent reviews and felt that they had received extra care; this made them feel that they were ‘taken seriously’ and they valued this support:

*‘I think they were a little bit more on the ball* […]*? I think they were more concerned about things. Because before you just went for your review and you were in and out in no time, but both these times I wasn’t in and out, so I felt that they were working on things better.’*

(P6, aged 69 years, asthma not well controlled)

Patients also had mixed views on the mode of delivery of asthma reviews. Those whose symptoms were well controlled seemed to favour a telephone review for its flexibility and convenience, whereas patients with less-controlled symptoms, or those wanting to discuss the feeling of being at risk and mental health concerns related to having asthma, would have preferred a face-to-face review:

*‘If you’re not having problems breathing and your asthma’s under control, maybe you know a phone call. But if my asthma was bad then I would then prefer to be seen properly.’*

(P8, aged 68 years, asthma well controlled)

The telephone review was seen as more rigid, and something that did not allow unscripted topics to be discussed:

*‘It was very detached and it was very much* [a] *ticking-the-box situation* […] *they just go through a number of standard questions. It is nice if we could see a face, and* […] *I say, if I go back to feeling a little bit vulnerable during this COVID business and some reassurance in certain areas about this would have been a bit more helpful if it was forthcoming.’*

(P2, aged 69 years, asthma well controlled)

In addition, patients often saw face-to-face reviews as the ‘gold standard’, as they enabled checking inhaler technique, discussions of medication, and doing peak flow tests.

#### Time 2

Similar to as at Time 1, some patients continued to have confidence that their GP practice would respond if they showed evidence of worsening asthma symptoms. They seemed to expect that reviews would happen if there were major changes to their condition, rather than as part of routine care. However, overall, there was more of an expectation that a review should have been offered by this point, and some highlighted that not having their medication reviewed for such a long time was worrying:

*‘Maybe that the dose I’m taking is not appropriate. It may be that the medication is not appropriate* […] *My peak flow was 240 this week, which isn’t great, but nobody knows that. It’s just like you’re doing it on your own really* […] *I would be pleased to go and be checked. She might examine my chest. She might discuss how I am or whatever, I don’t know but it would be reassuring really.’*

(P7, aged 76 years, asthma well controlled [last asthma review in 2019])

#### Time 3

By the time of the third interview, patients who had not been called for a routine review expressed their concerns about this, even if their symptoms were well controlled; this was particularly evident in patients who had some changes in their condition:

*‘I used to have at least an annual review of my medication, my peak flow and everything, and they would make suggestions about altering the dosage and maybe putting it up or down, whatever, or however I was but since I’ve been here, which is in 2019 I haven’t had any physical visits to the asthma nurse. So, I thought I was due for one and being out of breath I thought it was a valid request, but you can’t have one.’*

(P7, aged 76 years, asthma well controlled)

Among patients who had telephone or face-to-face reviews, some expressed the benefits of having a discussion about their asthma; however, some — for example, those who had not had a review for a while and had expectations of a more-comprehensive discussion that were not always met — also expressed dissatisfaction with their reviews:

*‘Yes, it* [review] *was a bit of an anti-climax* […] *I forgot to take my action plan, ‘cause I couldn’t find it* […] *but I wasn’t asked for it. And the nurse who carried it out basically just said, “How have you been? Have you had any problems?”, to which I said “no”, but I also asked if she’d check my blood pressure, which she did, and then she just said “Oh well, that’s fine then.” And I said “Could I possibly check my peak flow?” And she said “Oh no, we’re not using them at the moment because of COVID.”’*

(P9, aged 50 years, asthma controlled)

### The role of self-management strategies during the pandemic

#### Time 1

Due to often-limited access to reviews, patients described relying on known self-management strategies that had helped them pre-pandemic, such as avoiding their usual triggers, adjusting their medications if they needed it, keeping fit, or trying to improve their general health. However, they often highlighted their particular importance in the context of the pandemic, and some noted that they had been more diligent with their medication.

When patients were concerned about their asthma, they tried to make sense of any worrying changes by keeping a symptom diary or monitoring peak-flow measurements, if they had them at home. Patients with newly diagnosed asthma were particularly unsure how to monitor their condition and a minority highlighted poor access to inhalers in the initial stages of the pandemic, which had a negative impact on them:

*‘There was quite a large period of having to go without my inhaler because I couldn’t get an appointment* […] *It’s quite terrifying to be fair. You need medical support, whether it be by drugs or inhaler or whatever.’*

(P12, aged 39 years, asthma not well controlled)

Patients also reported pandemic-specific triggers, especially those related to stress and anxiety caused by the pandemic, including their uncertainties around their levels of risk. Consequently, they highlighted that they tried to use both new and previously used strategies to manage their mental health, such as breathing exercises, going for a walk, or generally looking after themselves; however, they also highlighted that the pandemic had been a particularly stressful time for them:

*‘It* [the pandemic] *plays on my mind and it’s stressful, and I’m absolutely convinced that this series of lockdowns and working from home and everything has really made people like me more sick than we should be. It’s not like, is there a correlation? No, this is bad for us.’*

(P5, aged 61 years, asthma well controlled)

In contrast, one patient noted that the pandemic had been a springboard for prioritising his health and making positive changes to his lifestyle that, in turn, had a positive impact on his asthma:

*‘I think this was the best period* [lockdown]*, for me personally, during my asthma, the whole asthma journey. It was quite beneficial just to slow down and exercise at the right time and focus on a good diet. It was nice just to relax, doing activities that you wouldn’t normally get the chance to do because of work.’*

(P11, aged 41 years, asthma well controlled)

#### Time 2

Patients described using the same strategies that had helped them during previous winters, including adjusting their medication, avoiding being exposed to cold air, and infection-control measures.

Compared with at Time 1, patients had various types of upper respiratory tract infections. Their first action often included increasing their medication to deal with symptoms such as breathlessness. Some felt unable to deal with their symptoms themselves and, consequently, were considering getting in touch with their practice, or had already done so, to seek advice:

*‘I’ve had it for a long time but it seems to have flared up a bit, probably because it’s been the wintertime and cold, going out for a run there’s been a few times where I’ve struggled to control it. I usually do about five kilometres, sometimes I can’t even do that because of the wheeze* […] *I have problems with Salbutamol, but I did take it a couple of times and once I was fine the next day and another time I felt a little bit more wheezy, but I have actually* […] *contacted the asthma nurse last Thursday to check.’*

(P4, aged 57 years, asthma well controlled)

For one person this resulted in ‘emergency’ reviews, with multiple follow-up appointments so that an asthma nurse could make changes to their medication.

#### Time 3

Patients continued using their self-management strategies, while waiting to be offered their review. When experiencing changes to their condition, some continued increasing or changing their medication, based on discussions with their health professionals and based on their own knowledge of their asthma:

*‘Yes, it’s something that I did based on how I feel. I’ve always kind of associated a cough with asthma, so I’ve always tried to treat it by increasing the number of puffs that I use.’*

(P4, aged 57 years, asthma well controlled)

Patients also highlighted the continued importance of looking after their mental and physical health, which came to the forefront during the pandemic. Feeling generally healthier, and using infection-control measures, were perceived as the main ways to be ready in case of asthma exacerbations and to prevent infections that could then, ultimately, affect their breathing.

## DISCUSSION

### Summary

This longitudinal qualitative interview study identified the dynamic nature of patients’ sense of risk, which was affected by multiple factors, and highlighted the need for a greater clarity about patients’ personal risk. Patients felt that routine asthma reviews should still have been conducted during the COVID-19 pandemic. As the pandemic progressed, they highlighted concern about limited access to opportunities to discuss their asthma with healthcare professionals. Patients with well-controlled symptoms felt that remote reviews were largely satisfactory, but still felt that face-to-face reviews were necessary for certain aspects of care, such as physical examination and patient-led discussion of sensitive or broader issues associated with asthma, including mental health.

### Strengths and limitations

This is the first study, to the authors’ knowledge, to explore in depth the experiences of patients with asthma in primary care over time in the context of the COVID-19 pandemic. The longitudinal design enabled the gathering of unique insights into changing views, needs, experiences, and expectations related to asthma management from the patients’ perspective. Conducting serial interviews also allowed researchers to build a rapport with patients over time,^31^ thereby potentially helping to provide rich data.

The study benefited from extensive discussions with a PPI panel, who were able to shape it by providing feedback on interview questions, making sense of data, and suggesting clinical implications. However, involving patients in the design of the study could have further enhanced the partnership with patient representatives.^32^^,^^33^

Despite a large number of interviews overall, and a varied age and sex profile among the sample, the study would have benefited from speaking with more patients whose symptoms were less controlled, as their experiences indicated some differences. Speaking to more of these patients might have enabled us to explore these differences in more detail.

### Comparison with existing literature

The results from Time 1 are in line with those of other studies, highlighting that patients were often unsure how much they were at risk, what being at risk meant in relation to their asthma, and why they would be classed as vulnerable, while also showing how — in the context of a lack of official classification — patients tried to understand their own risk status and often saw themselves as being at high risk.^34^^–^^36^ However, interviews conducted at later time points found that patients’ perception of risk was dynamic as they continuously assessed their risk, taking into account numerous factors, such as their asthma control, local incidence levels, their beliefs about the risk, severity of COVID-19 symptoms they or their close contacts had experienced, or being offered a booster vaccine. In addition, patients also highlighted a lack of information available about risk and seeking information themselves from online groups or charities.

In line with other studies,^2^^,^^37^^–^^40^ the study presented here showed that patients expressed diverse views on whether remote or in-person asthma reviews are most suitable for them. Remote care may offer convenience,^41^^,^^42^ improve access,^40^ increase attendance,^42^ and be a safe and acceptable alternative to face-to-face reviews,^42^ but it may not be suitable — or preferred — by all patients.^37^^–^^39^ In the study presented here, patients whose symptoms were not well controlled, who wanted to discuss their mental health or feelings of vulnerability, or who had not had contact with their GP practice for a long time, felt that face-to-face reviews would be preferable. In addition, face-to-face reviews were perceived as a ‘gold standard’, as they also allowed for the checking of inhaler technique and conducting of measurements such as peak flow. Incorporating these components remotely may be difficult for both patients and clinicians, with recent studies suggesting that clinicians may not feel able to educate, or knowledgeable about educating, patients on inhaler technique.^3^^,^^43^ Also, remote reviews were seen as more rigid, and inadvertently facilitated the scripted delivery of asthma reviews, rather than being patient led.

It has been highlighted elsewhere that appropriately trained multidisciplinary teams are essential to deliver high-quality asthma care and, thus, improve patient outcomes, which is challenging with increasing GP workloads and staff shortages.^44^ The study presented here also highlighted patients’ increasing concern about the scarcity of routine asthma reviews as the pandemic progressed. Patients’ concerns about lack of reviews seem to increase the longer they were not reviewed by their GP practice.

As highlighted by other studies, achieving asthma control is multifaceted and requires patients to engage in self-care behaviours, monitor symptoms, and actively engage with healthcare professionals.^45^ Given reports of limited primary care asthma reviews, patients seemed to rely on self-management; in line with the findings of another study,^46^ the authors found that patients relied on pre-existing self-management strategies, but newly diagnosed patients felt less confident at doing this, and some patients had experienced difficulties in accessing inhalers. In addition, some patients introduced new strategies, especially related to their mental health, as also highlighted by other studies.^47^

### Implications for research and practice

The study highlighted that patients’ perception of risk throughout the pandemic was dynamic, and not necessarily in line with the official guidance related to shielding, restrictions, or booster vaccinations. Specifically, some patients who were initially deemed as clinically vulnerable felt at continued risk, despite restrictions being lifted and advice on shielding being withdrawn, whereas patients who were not deemed at risk continued with strict social distancing. As evidence on whether asthma patients are at higher risk of infection or more-severe outcomes was unclear, discussions around patients’ views on their risk could be an important part of reviews.

Asthma reviews can be considered complex interventions composed of multiple components.^1^ While some components (including checking inhaler technique and discussing medication) are key in the context of the pandemic, broadening or tailoring the scope of asthma reviews to take into account features that are particularly relevant during a healthcare emergency are crucial. These can include discussions around dealing with stress and uncertainty, making sense of personal risk, and self-management strategies. Self-management strategies need to be discussed and updated through asthma reviews and action plans, which were not always available. Having regular discussions between patients and health professionals is crucial to understand patients’ perspectives^48^^,^^49^ and potential barriers to using their medications,^47^^,^^48^ which may be different from the clinicians’ perceptions.^50^ Although it may not be possible to always offer a face-to-face review, which patients believed facilitated discussing these issues, actively inviting discussion of such issues may be a way forward.

Given the limited contact with health professionals for some patients, the first review after the pandemic may be of a greater importance to patients than that held prior to the pandemic, and patients may expect healthcare professionals to make space for broader discussions around asthma and, generally, ‘taking stock’ of how they have been. This may be the case for other long-term conditions, in which annual reviews play a key part in their management in primary care. This could mean not only using the templates and protocols, for example, asthma action plans, as a starting point, but also complementing these by proactively enquiring about any areas that patients would like to discuss. This is particularly important in the context of increasing use of remote consultations, with recent data suggesting that nearly one-third of appointments in general practice still take place remotely.^51^

Asthma reviews are important to patients, even during periods when access to face-to-face consultations in primary care is more restricted than usual. These reviews, even if conducted remotely, should not only include monitoring of the patient’s asthma, but should also allow opportunities to address wider related issues, including mental health. Where possible, patients also need clearer guidance on their own personal risk of severe illness or complications from COVID-19 to inform their decisions about when, and how, to seek an asthma review; contradictory messages should be avoided.

Future research should explore ways of adapting reviews for patients with asthma, as well as those for patients with other chronic conditions during future pandemics; these adaptations should be feasible and allow appropriate precautions to be taken, while still meeting patients’ needs and expectations.

## References

[1] Stewart J, McCorry N, Reid H (2022). Implementation of remote asthma consulting in general practice in response to the COVID-19 pandemic: an evaluation using extended Normalisation Process Theory. BJGP Open.

[2] Wanat M, Hoste M, Gobat N (2021). Transformation of primary care during the COVID-19 pandemic: experiences of healthcare professionals in eight European countries. Br J Gen Pract.

[3] Papi A, Haughney J, Virchow JC (2011). Inhaler devices for asthma: a call for action in a neglected field. Eur Respir J.

[4] Bleecker ER, Al-Ahmad M, Bjermer L (2022). Systemic corticosteroids in asthma: a call to action from World Allergy Organization and Respiratory Effectiveness Group. World Allergy Organ J.

[5] Ryan D, Heatley H, Heaney LG (2021). Potential severe asthma hidden in UK primary care. J Allergy Clin Immunol Pract.

[6] Aaron SD, Boulet LP, Reddel HK, Gershon AS (2018). Underdiagnosis and overdiagnosis of asthma. Am J Respir Crit Care Med.

[7] Kaczorowski J, Del Grande C (2021). Beyond the tip of the iceberg: direct and indirect effects of COVID-19. Lancet Digit Health.

[8] Adams K, Greiner AC, Corrigan JM (2004). The 1st Annual Crossing the Quality Chasm Summit: a focus on communities. Report of a summit.

[9] Pinnock H, Parke HL, Panagioti M (2017). Systematic meta-review of supported self-management for asthma: a healthcare perspective. BMC Med.

[10] Hartmann-Boyce J, Gunnell J, Drake J (2021). Asthma and COVID-19: review of evidence on risks and management considerations. BMJ Evid Based Med.

[11] Department of Health and Social Care, Ministry of Housing, Communities and Local Government (2020). Guidance on shielding and protecting people who are clinically extremely vulnerable from COVID-19.

[12] Carli G, Cecchi L, Stebbing J (2021). Is asthma protective against COVID 19?. Allergy.

[13] Scott KM, Von Korff M, Ormel J (2007). Mental disorders among adults with asthma: results from the World Mental Health Survey. Gen Hosp Psychiatry.

[14] Fong WCG, Rafiq I, Harvey M (2022). The detrimental clinical associations of anxiety and depression with difficult asthma outcomes. J Pers Med.

[15] Smith SJ, Busby J, Heaney LG (2021). The impact of the first COVID-19 surge on severe asthma patients in the UK. Which is worse: the virus or the lockdown?. ERJ Open Res.

[16] Miles C, Arden-Close E, Thomas M (2017). Barriers and facilitators of effective self-management in asthma: systematic review and thematic synthesis of patient and healthcare professional views. NPJ Prim Care Respir Med.

[17] Khan N, Jones D, Grice A (2020). A brave new world: the new normal for general practice after the COVID-19 pandemic. BJGP Open.

[18] Verhoeven V, Tsakitzidis G, Philips H, Van Royen PJ (2020). Impact of the COVID-19 pandemic on the core functions of primary care: will the cure be worse than the disease? A qualitative interview study in Flemish GPs. BMJ Open.

[19] Ow NL, Sadek Attalla S, Davies G (2022). Experiences and behaviours of patients with asthma requesting prescriptions from primary care during medication shortages linked to the COVID-19-lockdown: insights from a qualitative analysis of a UK asthma online community. Br J Gen Pract.

[20] Arora N, Lowe D, Sarsour N (2022). Asthma care during COVID-19: differences in attitudes and expectations between physicians and patients. J Asthma.

[21] Caveney B, Halterman JS, Fagnano M (2022). Caregiver experiences managing persistent childhood asthma during the COVID-19 pandemic. Clin Pediatr.

[22] Jia Y, Bao J, Yi M (2021). Impact of the COVID-19 pandemic on asthma control among children: a qualitative study from caregivers’ perspectives and experiences. BMJ Open.

[23] Tyson L, Hardeman W, Stratton G (2022). The effects of social distancing and self-isolation during the COVID-19 pandemic on adults diagnosed with asthma: a qualitative study. J Health Psychol.

[24] Ekström S, Mogensen I, Georgelis A (2022). General stress among young adults with asthma during the COVID-19 pandemic. J Allergy Clin Immunol Pract.

[25] Neale B (2021). The craft of qualitative longitudinal research.

[26] Calman L, Brunton L, Molassiotis A (2013). Developing longitudinal qualitative designs: lessons learned and recommendations for health services research. BMC Med Res Methodol.

[27] Wanat M, Boylan A-M, Borek AJ (2021). Value, challenges and practical considerations when designing, conducting and analysing a longitudinal qualitative study in family medicine. Fam Med Community Health.

[28] NHS England, NHS Improvement (2021). Temporary GP contract changes to support COVID-19 vaccination programme.

[29] Grossoehme D, Lipstein E (2016). Analyzing longitudinal qualitative data: the application of trajectory and recurrent cross-sectional approaches. BMC Res Notes.

[30] Tong A, Sainsbury P, Craig J (2007). Consolidated criteria for reporting qualitative research (COREQ): a 32-item checklist for interviews and focus groups. Int J Qual Health Care.

[31] Grinyer A, Thomas C, Gubrium JF, Holstein JA, Marvasti AB, McKinney KD (2012). The value of interviewing on multiple occasions or longitudinally. The SAGE handbook of interview research: the complexity of the craft.

[32] Ryan D, Keighley A, Jackson T (2022). Patient perspectives in asthma: listening to and learning from a new paradigm in translational research. Respir Med.

[33] Jackson T, Pinnock H, Liew SM (2020). Patient and public involvement in research: from tokenistic box ticking to valued team members. BMC Med.

[34] Pedrozo-Pupo JC, Campo-Arias A (2020). Depression, perceived stress related to COVID, post-traumatic stress, and insomnia among asthma and COPD patients during the COVID-19 pandemic. Chron Respir Dis.

[35] Fisher A, Roberts A, McKinlay AR (2021). The impact of the COVID-19 pandemic on mental health and well-being of people living with a long-term physical health condition: a qualitative study. BMC Public Health.

[36] Philip KEJ, Lonergan B, Cumella A (2020). COVID-19 related concerns of people with long-term respiratory conditions: a qualitative study. BMC Pulm Med.

[37] Levene LS, Seidu S, Greenhalgh T, Khunti K (2020). Pandemic threatens primary care for long term conditions. BMJ.

[38] Wanat M, Hoste ME, Gobat NH (2022). Patients’ and clinicians’ perspectives on the primary care consultations for acute respiratory infections during the first wave of the COVID-19 pandemic: an eight-country qualitative study in Europe. BJGP Open.

[39] Greenhalgh T, Rosen R, Shaw SE (2021). Planning and evaluating remote consultation services: a new conceptual framework incorporating complexity and practical ethics. Front Digit Health.

[40] Pinnock H, Bawden R, Proctor S (2003). Accessibility, acceptability, and effectiveness in primary care of routine telephone review of asthma: pragmatic, randomised controlled trial. BMJ.

[41] Bradford NK, Caffery LJ, Smith AC (2016). Telehealth services in rural and remote Australia: a systematic review of models of care and factors influencing success and sustainability. Rural Remote Health.

[42] Kinley E, Skene I, Steed E (2022). Delivery of supported self management in remote asthma reviews: a systematic rapid realist review. Health Expect.

[43] Karle E, Patel TP, Zweig J, Krvavac A (2020). Understanding the knowledge gap and assessing comfort level among healthcare professionals who provide inhaler education. COPD.

[44] Fletcher MJ, Tsiligianni I, Kocks JWH (2020). Improving primary care management of asthma: do we know what really works?. NPJ Prim Care Respir Med.

[45] National Asthma Education and Prevention Program (2007). Expert Panel Report 3 (EPR-3): Guidelines for the Diagnosis and Management of Asthma — summary report 2007. J Allergy Clin Immunol.

[46] Chang C, Zhang L, Dong F (2021). Asthma control, self-management, and healthcare access during the COVID-19 epidemic in Beijing. Allergy.

[47] Tyson L, Hardeman W, Stratton G (2022). The effects of social distancing and self-isolation during the COVID-19 pandemic on adults diagnosed with asthma: a qualitative study. J Health Psychol.

[48] Horne R, Weinman J (2002). Self-regulation and self-management in asthma: exploring the role of illness perceptions and treatment beliefs in explaining non-adherence to preventer medication. Psychol Health.

[49] Horne R, Price D, Cleland J (2007). Can asthma control be improved by understanding the patient’s perspective?. BMC Pulm Med.

[50] Sapir T, Moreo KF, Greene LS (2017). Assessing patient and provider perceptions of factors associated with patient engagement in asthma care. Ann Am Thorac Soc.

[51] NHS Digital (2023). Appointments in general practice, December 2022. https://digital.nhs.uk/data-and-information/publications/statistical/appointments-in-general-practice/december-2022.

